# Methylation of the first exon in the erythropoietin receptor gene does not correlate with its mRNA and protein level in cancer cells

**DOI:** 10.1186/s12863-018-0706-8

**Published:** 2019-01-03

**Authors:** Barbora Fecková, Patrícia Kimáková, Lenka Ilkovičová, Erika Szentpéteriová, Mária Macejová, Ján Košuth, Anthony Zulli, Nataša Debeljak, Petra Hudler, Karin Jašek, Ivana Kašubová, Peter Kubatka, Peter Solár

**Affiliations:** 1 Department of Cell Biology, Institute of Biology and Ecology, Faculty of Science, Pavol Jozef Šafárik University in Košice, SK-04154 Košice, Slovak Republic; 2 Centre for Chronic Disease, College of Health & Biomedicine, Victoria University, Melbourne, Victoria Australia; 3 Institute of Biochemistry, Faculty of Medicine, University of Ljubljana, SI1000 Ljubljana, Slovenia; 4 Biomedical Centre Martin, Division of Oncology, Jessenius Faculty of Medicine in Martin, Comenius University in Bratislava, SK03601 Martin, Slovak Republic; 5 Department of Medical Biology, Jessenius Faculty of Medicine in Martin, Comenius University in Bratislava, SK03601 Martin, Slovak Republic; 6 Department of Experimental Carcinogenesis, Biomedical Centre Martin, Division of Oncology, Jessenius Faculty of Medicine in Martin, Comenius University in Bratislava, SK03601 Martin, Slovak Republic; 7 Institute of Medical Biology, Faculty of Medicine, Pavol Jozef Šafárik University in Košice, Trieda SNP 1, SK04011 Košice, Slovak Republic

**Keywords:** Erythropoietin receptor, Methylation, Transcript variants, Western blot, Cancer cells

## Abstract

**Background:**

Erythropoietin receptor (EPOR) is a functional membrane-bound cytokine receptor. Erythropoietin (EPO) represents an important hematopoietic factor for production, maturation and differentiation of erythroid progenitors. In non-hematopoietic tissue, EPO/EPOR signalization could also play cytoprotective and anti-apoptotic role. Several studies identified pro-stimulating EPO/EPOR effects in tumor cells; however, numerous studies opposed this fact due to the usage of unspecific EPOR antibodies and thus potential absence or very low levels of EPOR in tumor cells. It seems that this problem is more complex and therefore we have decided to focus on EPOR expression at several levels such as the role of methylation in the regulation of EPOR expression, identification of possible EPOR transcripts and the presence of EPOR protein in selected tumor cells.

**Methods:**

Methylation status was analysed by bisulfite conversion reaction, PCR and sequencing. The expression of EPOR was monitored by quantitative RT-PCR and western blot analysis.

**Results:**

In this study we investigated the methylation status of exon 1 of *EPOR* gene in selected human cancer cell lines. Our results indicated that CpGs methylation in exon 1 do not play a significant role in the regulation of *EPOR* transcription. However, methylation status of *EPOR* exon 1 was cell type dependent. We also observed the existence of two *EPOR* splice variants in human ovarian adenocarcinoma cell line - A2780 and confirmed the expression of EPOR protein in these cells using specific A82 anti-EPOR antibody.

**Conclusion:**

We outlined the methylation status of all selected cancer cell lines in exon 1 of *EPOR* gene and these results could benefit future investigations. Moreover, A82 antibody confirmed our previous results demonstrating the presence of functional EPOR in human ovarian adenocarcinoma A2780 cells.

## Background

The erythropoietin (EPO) is a glycoprotein produced by the kidney depending on the amount of oxygen [1]. During oxygen deprivation, heat shock protein 70 stimulates the production of EPO, which is the main regulator of erythropoiesis. EPO is involved in the formation, maturation and differentiation of erythroid precursors after binding to EPO specific membrane receptor (EPOR) [2]. Since the observed positive stimulating effect of EPO/EPOR on the haematopoiesis, the production of recombinant human EPO (rhEPO) was initiated by pharmaceutical companies and subsequently it was approved for anaemia treatment. Human rhEPO is also used as alternative to transfusion of blood in cancer patients where anaemia is accompanying sign of chemotherapy [3].

Human DNA is enriched by methylated cytosine residues (CpG; cytosine-phospho-guanosine dinucleotide) that have major functions in the epigenetic regulation of genes, cellular differentiation and are essential for normal development and genomic integrity [4]. Many human cancer cell lines harbour distinct variations in promoter CpG methylation patterns, which profoundly affect gene expression [5, 6] . Therefore, DNA methylation could serve as an epigenetic biomarker of human tumors. Methylation of the transcriptional initiation/elongation region is very important for transcriptional suppression. Methylation of the first exon is also tightly linked to transcriptional silencing of genes [7]. In addition, it was established that methylation of leading exon blocks gene transcription, whereas methylation of next downstream exons has been positively correlated with transcription initiated from upstream region. It was also postulated that first exon methylation could regulate the selection of alternative starts [8].

Splicing variants of *EPOR* were detected in the variety of cell lines and tumors [9]. Alternative splicing of *EPOR* results in three different *EPOR* transcripts with different hematopoietic function: full length EPOR (EPOR-F), truncated EPOR (EPOR-T) and soluble EPOR (EPOR-S). Introns between the seventh and the eighth exons are spliced to form EPOR-T with loss of part of the intracellular domain. EPOR-T was observed in normal hematopoietic tissue with apoptotic effects attenuating role in erythropoiesis and also in leukemic cells with proapoptotic and anti-apoptotic responses [10].

There are many studies demonstrating that EPO/EPOR signalization in cancer cells can: induce cell proliferation [11–14], change the sensitivity to chemotherapeutics [11, 12], induce angiogenesis [15] and/or tumor neovascularization [16]. However, there are studies where no growth response to EPO treatment was observed [17–19]. Furthermore, in some studies using a sensitive A82 anti-EPOR antibody no EPOR was detected or it was detected only in low levels in many different cancer cell lines [20, 21]. These facts lead to additional questions; the most important of which is, what could be the reason for such variations in outcomes from different studies. Could these differences be attributed to methodological procedures, sources of cell lines or usage of the different (possibly non-specific) antibodies? In this regard, we adopted the opinion of Patterson [22], that the differences in studies are mainly the consequence of the distribution of unspecific primary EPOR antibodies. As a result, not only the presence of EPOR protein, but also its amount or its size differs in the observed cell lines [23].

In our study, we focused on the monitoring of CpG sites around the first exon (+ 1/+ 125) of *EPOR* gene (NG_021395.1) in various cancer cell lines because of large *EPOR* promoter homogeneity with other genes and very high homogeneity and tandem repetitions in *EPOR* promoter itself. We decided to search for potential correlation between the methylation status in this region and its transcriptional activity as well as EPOR spliced variants. EPOR protein level in all monitored cell lines was evaluated using three different antibodies.

## Methods

### Cell culture conditions

The ovarian adenocarcinoma A2780, lung adenocarcinoma A549, colorectal adenocarcinoma HT-29, hepatocellular carcinoma HEP-G2, mammary adenocarcinoma MCF-7 and mammary carcinoma T47D cell lines were obtained from the American Tissue Culture Collection (ATCC; VA, USA). The acute myeloid leukemia UT-7 cells and renal carcinoma 769P cell lines were purchased from Leibniz - Institut DSMZ, Deutsche Sammlung von Mikroorganismen und Zellkulturen GmbH (DSMZ; Germany). The parental non-metastatic benign tumor-derived rat mammary epithelial cells RAMA 37 and its derived stably transformed cell subclone RAMA 37–28 [24], transfected with pcDNA3.1 expression vector contained wild type human *EPOR* gene [using 1.0 mg/ml geneticin selection of modified cells [25]] were obtained as a gift from University of Ljubljana, Faculty of Medicine. All cell lines were grown in RPMI-1640 medium supplemented with L-glutamine (Gibco; Thermo Fisher Scientific, Inc., MA, USA), 10% fetal bovine serum (Gibco; Thermo Fisher Scientific, Inc.) and antibiotic and antimycotic solution (100 U/ml penicillin - 100 μg/ml streptomycin and 0.25 μg/ml amphotericin B; Thermo Fisher Scientific, Inc.). The medium for UT-7 cell line was enriched with 1 U/ml rhEPO (40,000 U/ml; EPREX®; Janssen Biologics B.V., Netherlands) and the medium for T47D cells with 100 U/ ml Insulin (1: 1,000; Humulin M3 Cartridge; Lilly France S.A.S., France)**.** We used standard cell culture conditions with 37 °C and 5% CO_2_/95% air and ZF Coulter counter (Beckman Coulter, Inc., CA, USA) for determination of cells number. Additionally, cell viability using 0.15% eosin staining and the light microscopy was analysed.

### Methylation analysis

#### DNA isolation

Genomic DNA (gDNA) from cell lines was isolated according to the protocol by GeneJET Genomic DNA Purification Kit (K0721; Thermo Fisher Scientific, Inc.). DNA samples were quantified by spectrophotometer BioSpec-nano (Shimadzu Scientific Instruments, MD, USA) and the integrity was analysed by horizontal 1.2% agarose gel electrophoresis stained with GelRed Nucleic Acid (Biotium, Inc., USA).

#### Bisulfite conversion reaction

Bisulfite modification was performed by MethylCode™ Bisulfite Conversion Kit (MECOV-50; Invitrogen, CA, USA). After the conversion, 500 ng (200 ng for positive control) of DNA was diluted in 10 μl of Elution buffer.

#### Acquisition of fully methylated genomic DNA

For higher yield of fully methylated DNA (positive control), the gDNA (1 μg) was subjected to restriction by Bam*HI* endonuclease (40 U / μl; 10,798,975,001; Roche Applied Science, Germany) with 10 X SuRE/Cut Buffer in total volume of 50 μl at 37 °C / overnight. Inactivation of Bam*HI* was performed for 15 min at 65 °C. The cleavage of DNA samples was then verified by horizontal 1% agarose gel electrophoresis. The enzyme and buffer compounds were removed by ethanol precipitation. Briefly, DNA was mixed with 0.1 volumes of 3 M Sodium acetate (pH 5.2, final concentration 0.3 M) and 3 volumes of ice cold 100% ethanol and incubated for 1 h at − 80 °C. After precipitation, DNA pellets were centrifuged (13,000 x g / 30 min / 4 °C), washed twice with ice cold 75% ethanol, followed by resuspension of air-dried DNA in nuclease free water and measured concentration. Fully methylated DNA was prepared by using CpG methyltransferase (M.SssI; New England Biolabs, Inc., UK). For adequate amount of DNA, we performed methyltransferation in triplicate (one tube / 100 ng of DNA). The 100 ng of DNA was protected by 0.04 U / μl of M.SssI, 160 μM S-adenosylmethionine (SAM) and 1 X NEBuffer 2 in total volume 20 μl until 1 h at 37 °C. For enzyme inactivation and removing S-adenosyl-L-homocysteine (SAH, inhibiting product from SAM), we performed ethanol precipitation. After that, the same DNA was protected by M.SssI and ethanol-purified again. Fully methylated DNA (< residual 200 ng) was subjected to bisulfite conversion as described above.

#### Primer design and PCR conditions

The *EPOR* promoter and gene sequence (NG_021395.1) was analysed by MethPrimer software (http://www.urogene.org/methprimer/) to identify CpG sites. After this analyse, we designed primers for sequence around first exon in normal gDNA (forward primer 5’-CTG GTC GGG AAG GGC CTG GTC AGC T-3′, reverse primer 5’-CAC GCA GCT CAT CCT TAC CTT TGC TCT CGA ACT TGG-3′) and bisulfite modificated DNA (forward primer 5′-ATT TGT TAT TTA GAG GCG TTT GGT CGG GAA GG-3′, reverse primer 5’-CCA CAC GCA ACT CAT CCT TAC CTT TAC TCT C-3′). PCRs with normal unmethylated gDNA (negative control), bisulfite modificated DNA and fully methylated bisulfite modificated DNA (positive control) were performed in duplicates by C1000 Touch Thermal Cycler (Bio-Rad Laboratories, Inc.), respectively. A 50 μl reaction volume contained 0.025 U / μl of AmpliTaq Gold 360 DNA polymerase, 10 X AmpliTaq Gold 360 Buffer, 1.5 mM MgCl_2_, 2.6 μl of GC Enhancer or 1.3 μl of GC Enhancer (bisulfite modificated DNA and positive control), 0.5 μM forward and reverse primer and 83 ng of gDNA. The reaction conditions were: denaturation 10 min at 95 °C, 30 cycles, consisting of denaturation for 30 s at 95 °C, annealing for 30 s at 68.5 °C or 62 °C (bisulfite modificated DNA and positive control) and extension for 20 s at 72 °C, following by final extension for 7 min at 72 °C. PCR products were then subjected to electrophoresis in 1.5% agarose gel stained with GelRed Nucleic Acid (Biotium, Inc.).

#### Sequencing

The PCR products were purified using ExoSAP-ITTM PCR Product CleanUp Reagent (Thermo Fischer Scientific, Inc.) for 15 min at 37 °C followed by incubation for 15 min at 85 °C. After agarose gel electrophoresis control stained with GelRed Nucleic Acid (Biotium, Inc.), we performed sequencing reaction (10 μl) using sequencing kit BigDye Terminator v1.1 (Applied Biosystems, Inc., CA, USA). The reaction mixtures for normal DNA (negative control) contained 1 μl of BigDye mix (dNTPs, ddNTPs, DNA polymerase), 1 μl of 10 μM forward primer for normal DNA, 0.5 μl of diluted PCR product and 7.5 μl of dH_2_O. The reaction mixtures for bisufite modificated DNA or positive control contained 2 μl of BigDye mix, 1 μl of 10 μM forward primer for bisufite DNA, 1 μl of PCR product and 6 μl of dH_2_O. The PCRs were performed in Personal Thermal Cycler MJ Mini (Bio-Rad Laboratories, Inc.). The reaction conditions were: 2 min at 95 °C, 35 cycles, consisting of denaturation for 15 s at 95 °C, annealing for 30 s at 68.5 °C (negative control) or 62 °C (bisulfite modificated DNA and positive control) and extension for 4 min at 60 °C, followed by final extension for 7 min at 60 °C. After that, the 10 μl of PCR products were purified by SigmaSpin Post-Reaction Clean-Up Columns (Sigma-Aldrich, Inc., MO, USA). For capillary electrophoresis, we prepared a mixture of 12 μl of Formamid (HiDi Formamide, Applied Biosystems, USA) and 3 μl of purified PCR products in sequencing microplate. The samples in microplate were denatured for 3 min at 95 °C and then cooled down on ice. The sequencing analyses proceeded in 3500 Genetic Analyser (Applied Biosystems, Inc.) by capillary electrophoresis (3500 Capillary Array, Applied Biosystems, Inc.) and separated polymer (POP-7™ Polymer for 3500/3500xL Genetic Analysers, Applied Biosystems, Inc.). The obtained sequences we analysed by SnapGene Viewer (http://www.snapgene.com) and aligned with sequences in database BLAST (http://blast.ncbi.nlm.nih.gov/Blast.cgi).

### RNA isolation, reverse transcription and quantitative RT-PCR

The RNA was isolated using TRIzol™ reagent (Gibco, Invitrogen, NY, USA) according to the manufacturer’s instructions. The concentration of RNA was determined at 260 nm and purity was controlled by the ratio 260/280 nm and 260/230 nm using spectrophotometer BioSpec-nano (Shimadzu Scientific Instruments). The integrity and quality of RNA was verified by horizontal 1% agarose gel electrophoresis. Then, 1 μg of RNA was reverse transcribed using mixture of oligo(dT) and random primers by iScript™ Advanced cDNA Synthesis Kit for RT-qPCR (Bio-Rad Laboratories, Inc., CA, USA). Quantitative RT-PCRs (each analysis in duplicate) were done using CFX96 Touch Real Time PCR Detection System (Bio-Rad Laboratories, Inc.) in 10 μl of reaction volume containing 1 X iTaq™ Universal SYBR Green Supermix, 0.5 μM forward and reverse primers and 1 μl of cDNA. We used PCR conditions as it is follows: 30 s at 95 °C, 40 cycles of denaturation 5 s at 95 °C, annealing / extension 45 s at 55.5 °C, 61 °C or 60 °C for *EPOR-F*, *EPOR-T* or *EPOR-S*, respectively. The amplification was followed by melting curve analysis to confirm amplification of the desired single and specific product. *EPOR-F* primers (forward primer: 5’-GCT GGA AGT TAC CCT TGT GG-3′, reverse primer: 5’-CTC ATC CTC GTG GTC ATC CT-3′; the amplicon length: 148 bp) were designed by Trošt et al. [26], *EPOR-T* primers (forward primer: 5’-CTG ACG CCT AGC GAC CTG GAC C-3′, reverse primer: 5’-GCA GTT TGG CTG CAA GAA GCA-3′; the amplicon length: 249 bp) and *EPOR-S* primers (forward primer: 5′-GGA GCC AGG GCG AAT CAC GG -3′, reverse primer: 5′- GCC TTC AAA CTC GCT CTC TG -3′; the amplicon length: 204 bp) by Arcasoy et al. [9]. The expression of the tested genes was normalized to the expression of internal reference gene (*β actin*). The relative expression of *EPOR* and *β actin* (forward primer: 5’-ACC AAC TGG GAC GAC ATG GAG AAA ATC-3′, reverse primer: 5′-GTA GCC GCG CTC GGT GAG GAT CTT CAT-3′; the amplicon length: 366 bp) were obtained through the calculation of standard curves with cDNA mixtures (diluted four-fold times) used. The relative *EPOR* expression was normalized to the expression of *β actin*. The results are presented as mean ± standard deviation of three independent experiments. Only samples with Ct values ≤28 were considered for quantification (*EPOR-F* and *EPOR-T* in all tested samples). The samples with Ct values > 28 acquired the amplification product bellow the limit as UQL (under quantification limit).

### EPOR protein detection

All cell lines were washed twice with 1 X ice-cold phosphate-buffered saline (PBS; pH 7.2–7.4), scraped into lysis buffer [Tris-HCl (pH 7.4), 0.1% SDS, 10% glycerol and 100 X protease inhibitor cocktail (Sigma-Aldrich, Inc.)] and incubated for 45 min. Then, lysates were homogenized by sonication on ice for 30 s at 30 V (Sonopuls HD 2070; Bandelin electronic GmbH & Co. KG, Germany). After sonication, lysates were centrifuged at 10,000 x g for 10 min at 4 °C and the supernatants were transferred into 1.5 ml microcentrifuge tubes and quantified according to the Lowry protein assay protocol (Bio-Rad Laboratories, Inc.). Lysates (100 μg or 300 μg each) were then boiled in 4 X Laemmli Sample buffer (1,610,747; Bio-Rad Laboratories, Inc.) for 10 min, separated with 12% SDS-polyacrylamide gel electrophoresis (PAGE) and blotted onto a polyvinylidene fluoride (PVDF) membranes with transfer buffer [3.6 g Tris, 18 g glycine and 10% methanol (pH 7.4)]. The PVDF membranes were washed for 10 min with 1 X PBS (pH 7.2–7.4) or 1 X Tris-buffered saline (TBS; pH 7.2–7.4) and blocked with 5% non-fat milk in 1 X PBS + 0.1% Tween [1X PBST; pH 7.2–7.4 (Sigma-Aldrich, Inc.)] or 1 X TBS + 0.1% Tween (1 X TBST; pH 7.2–7.4) for 45 min. Then, the membranes were washed for 1 min and incubated overnight at 4 °C with primary anti-EPOR monoclonal antibodies respectively: A82 **(**1: 1250; Amgen, Inc., CA, USA) with 1% non-fat milk in 1 X TBST (pH 7.2–7.4), AF322PB (1: 500; R&D Systems, Inc., MN, USA) and AT1931a (1: 5000; Abgent, Inc., CA, USA) with 1% non-fat milk in 1 X PBST (pH 7.2–7.4). As loading control, we used primary anti-ß actin antibody (8H10D10; 1: 1000; Cell Signaling Technology, Inc., Leiden, Netherlands) in 5% non-fat milk in 1 X TBST (pH 7.2–7.4).

Next day, the membranes were washed three times for 10 min in 1 X TBST (pH 7.2–7.4) or 1 X PBST (pH 7.2–7.4) and incubated for 1 h with secondary horseradish peroxidase (HRP) - conjugated antibodies: goat anti-rabbit (31,461; 1: 5000; Thermo Fisher Scientific, Inc.), rabbit anti-goat (31,403; 1: 10,000; Thermo Fisher Scientific, Inc.) and goat anti-mouse (31,436; 1: 10,000; Thermo Fisher Scientific, Inc.) at room temperature (RT). The antibody reactivity was visualized with Pierce ECL Western Blotting Substrate (Thermo Fisher Scientific, Inc.). Bioluminescent signals were detected with ChemiDoc™ XRS+ and Image Lab 3.0 software (Bio-Rad Laboratories, Inc.) or X-ray films (Roberts Technology Group, Inc., PA, USA).

## Results

### Detection of CpG sites methylation in *EPOR* first exon by dideoxy sequencing

**Fig. 1:**
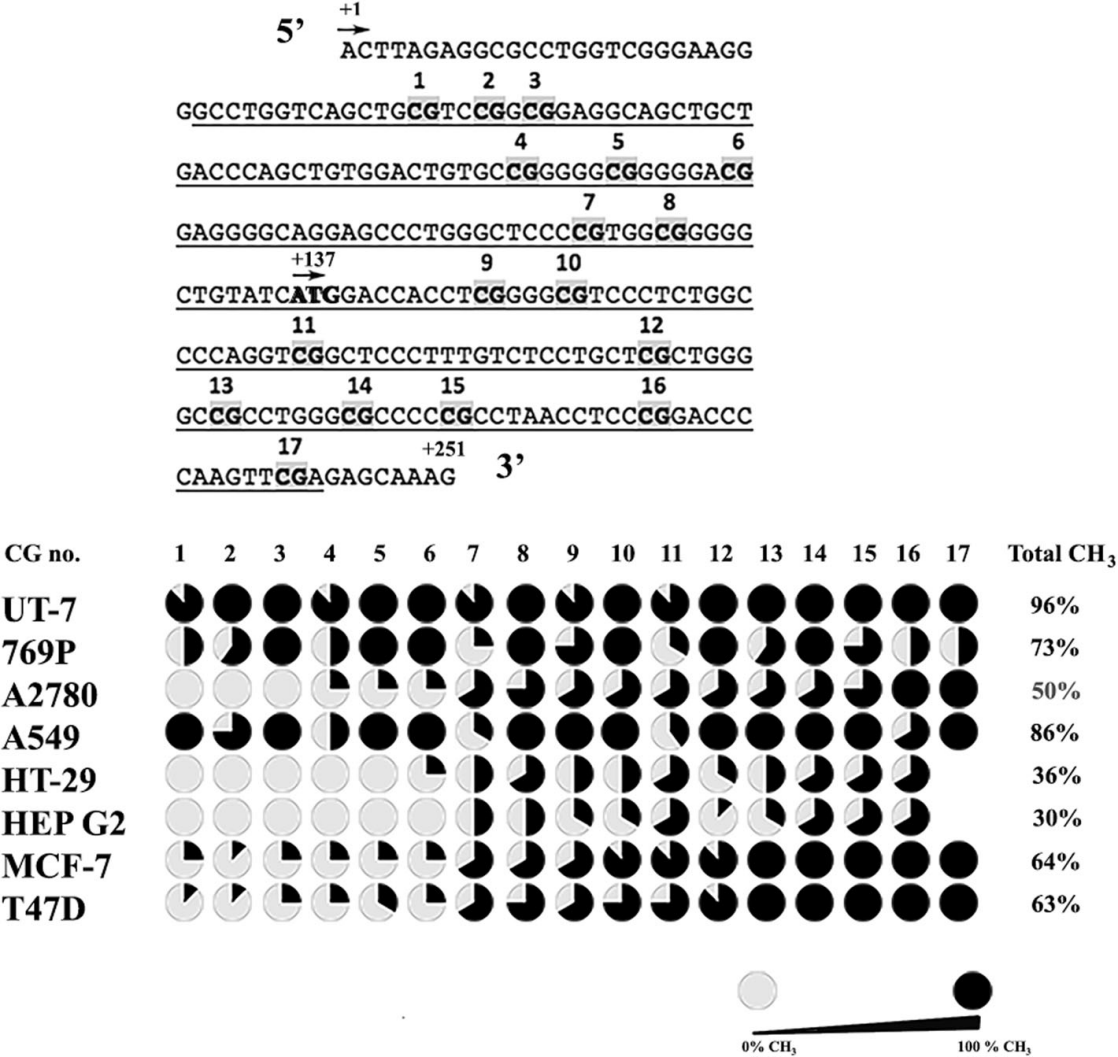
The methylation status of the first exon of *EPOR* gene in eight human cancer cell lines. The upper figure presents the sequence of *EPOR* first exon (+ 1/+ 251), the transcriptional start site (+ 1), the translation start site (+ 137), bold grey 17 CpG dinucleotides and the underlined monitored part. The lower part of figure demonstrates the methylation status of the first exon of *EPOR* gene. The pie charts are depicting the percentage of methylation, where black color represents full methylation of CpG dinucleotides and grey color represents unmethylated CpG. TSS, transcriptional start site; CG, cytosine-guanosine dinucleotide; EPOR, erythropoietin receptor

We defined CpG sites in *EPOR* promoter and gene body with MethPrimer software. Because of large *EPOR* promoter homogeneity with other genes and very high homogeneity and tandem repetitions in *EPOR* promoter itself, the area around first exon was chosen. We performed bisulfite modification of the DNA isolated from eight human cancer cell lines. Bisulfite modification discriminates between cytosine and methylated cytosine by bisulfite salt conversion of cytosine to uracil while methylated cytosine remains unchanged. After dideoxy sequencing of normal DNA (negative control), bisulfite modified DNA (experimental group) and fully methylated DNA (positive control) sequences were compared. The comparison of bisulfite modified DNA isolated from eight human cancer cell lines (UT-7, 769-P, A2780, A549, HT-29, HEP G2, MCF-7, T47D) is presented in Fig. 1. *EPOR* positive cancer cell line UT-7 revealed the highest 96% methylation profile of monitored sequence. Very high methylation was also observed in A549 cell line (86%) and in EPOR negative cancer cell line 769P (73%). Interestingly, human breast cancer cell lines MCF-7 and T47D demonstrated similar high 64 and 63% methylation profile, respectively. The medium methylation of the exon 1 of *EPOR* was observed in A2780 cell line (50%), whereas HT-29 and and HEP G2 cell lines showed the lowest methylation levels, 36 and 30%, respectively.

### Detection of *EPOR-F* and *EPOR-T* splicing variants

**Fig. 2:**
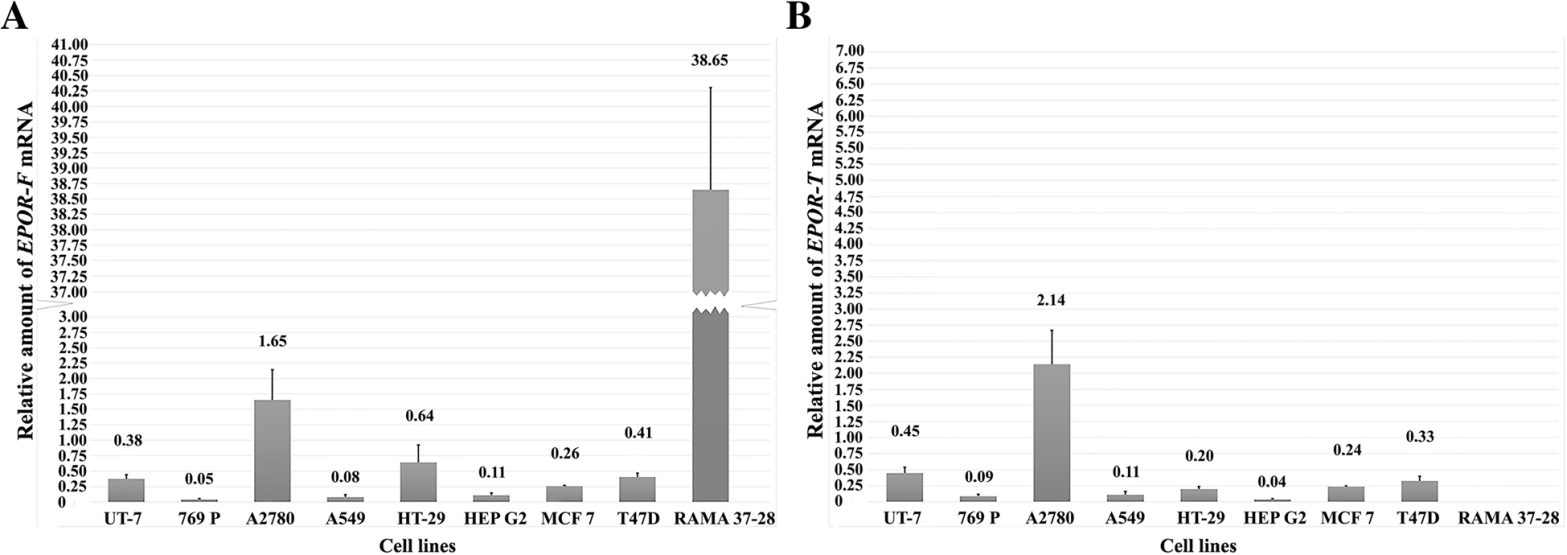
The relative amount of *EPOR-F* (**a**) and *EPOR-T* (**b**) mRNA transcripts in cancer cell lines. The expression of both *EPOR-F* and *EPOR-T* were normalized to *ß actin* expression. Data are presented as means ± SD of three independent experiments. EPOR-F, full length erythropoietin receptor; EPOR-T, truncated erythropoietin receptor

Based on our finding that *EPOR* positive UT-7 cells did not reveal expected strong expression, we selected a new positive control. In this regard, RAMA 37–28 - stably transformed cell line with wild type human *EPOR* gene was used. The expression of splicing variants of *EPOR* gene was normalized to *ß actin* expression. The relative values are shown in Fig. 2. In addition to high expression of variant *EPOR-F* in RAMA 37–28 cell line (38.65), we also observed expression of this variant in A2780 cell line (1.65). Compared to A2780 cells low expression of *EPOR-F* was found also in all monitored cancer cells (UT-7 0.38, 769P 0.05, A549 0.08, HT-29 0.64, HEP G2 0.11, MCF-7 0.26 and T47D 0.41). For details see Fig. 2a. Interestingly, A2780 cells demonstrated higher expression of *EPOR-T* (2.14) than *EPOR-F* splicing variant. Very low expression of *EPOR-T* was also seen in other tested human cancer cell lines (UT-7 0.45, 769P 0.09, A549 0.11, HT-29 0.20, HEP G2 0.04, MCF-7 0.24 and T47D 0.33) (Fig. 2b).

*EPOR-S* mRNA transcript variant was weakly detected in UT-7, A2780 and A549 cell lines, whereas the rest of analysed human cancer cell lines (769P, HT-29, HEP G2, MCF-7 and T47D) revealed only the traces of *EPOR-S* mRNA. Therefore, the quantification was unreliable and assigned to belong under quantification limit (UQL) (data not shown).

### EPOR protein detection

**Fig. 3:**
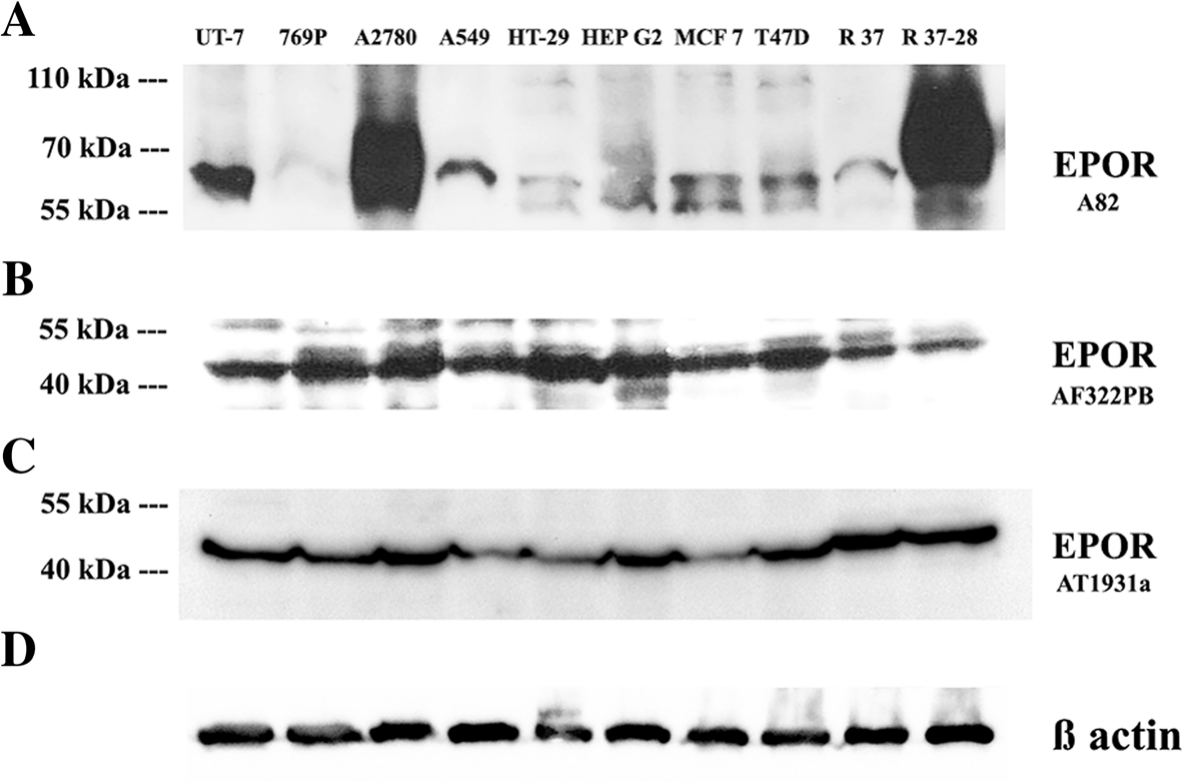
Western blot analysis of cell lysates using three anti-EPOR primary antibodies: A82 in size < 59 kDa (**a**) and AF322PB (**b**), AT1931a in size < 47 kDa (**c**). The detection of ß actin protein served as loading control (**d**). EPOR, erythropoietin receptor; kDa, kilo Dalton; R 37, RAMA 37; R37–28, RAMA 37–28

The controversial specificity of primary monoclonal anti-EPOR antibodies available on the market, lead us to test three of available antibodies (Fig. 3). Western Blot analysis with A82 antibody (donated by Amgen, Inc.) confirmed high expression of EPOR protein (< 59 kDa) in RAMA 37–28 as well as A2780 cell lines, which correlated with high expression of *EPOR* gene in those cells (Fig. 3a). A smaller amount of EPOR protein was detected in UT-7 and other cell lines. The only exception was *EPOR* negative 769P cell line, showing no EPOR signal. Interestingly, in comparison with A82 antibody, AF322PB and the AT1931a antibodies detected approximately equal EPOR signals in all cell lines, however, the positive signal was observed at approximately 47 kDa (Fig. 3b and c). From these observations, we could conclude that distinct signals, detected by AF322PB and AT1931a antibodies, could be due to non-specificity of these two tested antibodies. For all comparisons, the anti-ß actin antibody was used as a loading control (Fig. 3d).

## Discussion

Epigenetic inactivation of tumor suppressor coding and non-coding genes in human cancer and its role in aberrant division, immortality, genomic instability, metastasis and metabolic reprogramming of tumor cells have been extensively reviewed in the paper of [27]. Based on demethylation of genomic DNA, resulting in upregulation of *EPOR* mRNA Wallach et al. [28] hypothesized that *EPOR* downregulation during brain development could be a result of epigenetic alterations. According to the same authors [28] there are 31 CpGs in EPOR 5′-flanking region from nt − 1779 to − 606 followed by 330 bp long CpG-free sequence. Another sequence of 19 CpGs was found at the position − 274 nt going up to the translation start site (+ 137).

The methylation of promoter regions is well described; however, the role of open reading frame methylation is still unclear. In this study we decided to evaluate the methylation status of *EPOR* gene in the region of first exon. Jones [29] suggested that the position of the methylation patterns in the transcription units could have varying effects on the gene expression. In this regard, methylation in the close vicinity of the TSS blocks initiation of transcription. Singer et al. [30] demonstrated on human fibroblast cell-line and primary B cells that intragenic methylation correlates well with gene expression and that exons are more highly methylated than their neighbouring introns. Recently, Song et al. [31] confirmed the existence of positive correlation between exon-level DNA methylation status and mRNA expression in the Pacific oyster of *Crassostrea gigas.*

The majority of our selected cancer cell lines, with the exception of A2780 cell line, showed negative correlation between the monitored methylation of *EPOR* CpG sites and its transcription. Indeed, A2780 cell line was the only cancer cell line, where 50% methylation rate of observed CpG sites did not negatively influence the expression of *EPOR.* On the contrary, the expression of *EPOR* was higher in A2780 compared to *EPOR* positive UT-7 cancer cells. Interestingly, the rate of methylation in cell lines (A2780, HT-29, HEP G2, MCF-7, T47D) was less intensive in the first six CpG sites than in the rest of eleven ones (last nine CpGs are part of exon 1) which in TSS sequence correspond to the results obtained by Wallach et al. [28]. In contrast, the rate of methylation in cell lines UT-7, 769P and A549 was relatively high across all examined CpG sites. Wallach et al. [28] revealed low methylation rate and a different methylation pattern of CpGs in − 300/+ 149 fragment of *EPOR* comparing SY-SY5Y cells (fetal neuronal phenotype) to specimens of human adult brain. Moreover, the methylation in mentioned sequence did not totally reduce *EPOR* transcription [28]. We have observed a similar trend, as despite of high methylation rate some of our cells demonstrated low *EPOR* transcription. In this regard, the highest methylation status of our monitored sequence (96%) found in *EPOR* positive UT-7 cells did not inhibit the transcription of *EPOR* gene, and low concentrations of mRNA were detected. However, in *EPOR* negative 769P cancer cell line, high 73% rate of methylation inhibited the transcription of *EPOR*. Based on this contradictory result, we could conclude that the methylation of CpGs in exon 1 of *EPOR* gene does not play a significant role in the regulation of *EPOR* transcription. Nevertheless, it appeared that the methylation status of this area in *EPOR* gene could be cell type dependent. For example, human breast cancer cell lines MCF-7 and T47D revealed similar CpG methylations in this region of *EPOR* gene, which is also a feature, which differentiates these cell lines from the others. In this regard, Brenet et al. [7] proposed that genes with the lowest transcription levels have specific methylated regions in the first exons. Moreover, the DNA methylation of genomic regions close to TSS and first exon was strongly associated with gene repression, and interestingly, the effects of the methylation patterns in these regions on gene expression were different in different molecular subtypes of breast cancer [7].

It has been suggested that the methylation of gene bodies might even stimulate transcription elongation and/or influence the splicing of the genes [29]. Recently, Song et al. [31] demonstrated an association between exon-level DNA methylation and mRNA expression in the oyster and suggested also that the exon-level DNA methylation might play a role in the alternative splicing by positively affecting exon inclusion during transcription [31].

We have evaluated mRNA levels of two transcription forms of *EPOR, EPOR-F* and *EPOR-T.* We compared mRNA of these two splicing variants in *EPOR* positive cell lines as well as in negative control; however, we did not observe correlation between the methylation status of CpGs in exon 1 and the occurrence of full length or truncated mRNA transcripts of *EPOR*. In comparison with all selected cell lines, a slightly increased amount of truncated variant of *EPOR* was detected in A2780 cell line. In order to identify the correlation between epigenetic regulation and splicing patterns of *EPOR*, further studies are needed.

To demonstrate the protein levels of *EPOR* gene in cell lines we used recommended specific anti-EPOR antibody – A82 [23] for EPOR detection in non-erythroid cells. The A82 antibody was optimized for flow cytometry as well as western blot detection and the size of the protein detected by western blot analysis was 59 kDa. The authors found out that the positive transcription signals of *EPOR* positive control cells were proportional to EPOR protein level with a minimal signal of *EPOR* expression in negative cells [23]. In this study, we compared three commercially available EPOR antibodies, A82, AF322PB and AT1931a. Our results confirmed the specificity of A82 antibody for the detection of EPOR protein in EPOR positive UT-7 and EPOR overexpressing RAMA 37–28 cell lines. However, AF322PB and AT1931a antibodies did not show the desired specificity. Surprisingly, in cell line A2780 we observed stronger expression of *EPOR* than in positive control cell line UT-7, both at mRNA as well as at protein level (A82 antibody). The discrepancy in *EPOR* expression in A2780, UT-7 and other cell lines might be the consequence of different culturing (inactivated or regular serum) and/or experimental conditions [32]. In our studies, we usually use A2780 cells descended from ATCC and we analyse them between the passages 23–25 using standard RPMI media and an inactivated serum (see Material and methods section). Nevertheless, using A82 antibody we confirmed our previous results demonstrating the presence (expression) of the functional EPOR in this particular A2780 cell line [33–35]. In addition, we outlined the methylation status of all selected cancer cell lines in exon 1 of *EPOR* gene and these results could benefit future investigations of the significance of the methylation in the vicinity of the first exon and its relation to the transcriptional and/or splicing variant regulation.

## Conclusion

However the methylation status of *EPOR* exon 1 was cell type dependent CpGs methylation in this exon do not play a significant role in the regulation of *EPOR* transcription. We also demonstrated the existence of two *EPOR* splice variants in human ovarian adenocarcinoma cell line - A2780 and confirmed the expression of EPOR protein in these cells using specific A82 anti-EPOR antibody.

## Abbreviations

CpG: Cytosine-phospho-guanosine dinucleotide
EPO: Erythropoietin
EPOR: Erythropoietin receptor
EPOR-F: Full length EPOR
EPOR-S: Soluble EPOR
EPOR-T: Truncated EPOR
PAGE: Polyacrylamide gel electrophoresis
PBS: Phosphate-buffered saline
PVDF: Polyvinylidene fluoride
rhEpo: Recombinant human EPO
SAH: S-adenosyl-L-homocysteine
SAM: S-adenosylmethionine
TBS: Tris-buffered saline
UQL: Under quantification limit
